# Artificial Pancreas Device Systems for the Closed-Loop Control of Type 1 Diabetes: What Systems Are in Development?

**DOI:** 10.1177/1932296815617968

**Published:** 2015-11-20

**Authors:** Sara Trevitt, Sue Simpson, Annette Wood

**Affiliations:** 1 NIHR Horizon Scanning Research & Intelligence Centre, University of Birmingham, Birmingham, UK

**Keywords:** algorithm, artificial pancreas, closed-loop control, device system, glycemic control, type 1 diabetes

## Abstract

**Background:**

Closed-loop artificial pancreas device (APD) systems are externally worn medical devices that are being developed to enable people with type 1 diabetes to regulate their blood glucose levels in a more automated way. The innovative concept of this emerging technology is that hands-free, continuous, glycemic control can be achieved by using digital communication technology and advanced computer algorithms.

**Methods:**

A horizon scanning review of this field was conducted using online sources of intelligence to identify systems in development. The systems were classified into subtypes according to their level of automation, the hormonal and glycemic control approaches used, and their research setting.

**Results:**

Eighteen closed-loop APD systems were identified. All were being tested in clinical trials prior to potential commercialization. Six were being studied in the home setting, 5 in outpatient settings, and 7 in inpatient settings. It is estimated that 2 systems may become commercially available in the EU by the end of 2016, 1 during 2017, and 2 more in 2018.

**Conclusions:**

There are around 18 closed-loop APD systems progressing through early stages of clinical development. Only a few of these are currently in phase 3 trials and in settings that replicate real life.

Wearable medical devices that can be used for the continuous management of type 1 diabetes are already available, namely continuous glucose monitors (CGMs) and insulin pumps to deliver continuous subcutaneous insulin infusion (CSII) therapy. In addition, 2 types of non-closed-loop artificial pancreas device (APD) systems (ie, not fully automated) systems are available: low glucose suspend and predictive low glucose suspend devices (known as “first-generation” APD systems). Research to date suggests that APD systems may be better than CSII therapy alone in terms of increased time within target blood glucose range, reduced frequency of hypoglycemia and better overnight control.^1-3^ APDs may therefore offer a new largely automated way of managing type 1 diabetes more easily and effectively in the near future.

The focus of this article is a group of more complex APD systems that are defined as being “closed-loop,” meaning that they are fully automated. Closed-loop APDs are externally worn medical device systems that combine 3 functions: (1) the monitoring function carried out by a CGM, which is connected wirelessly to (2) the hormone therapy delivery function carried out by a pump, and (3) a digital controller (the “brain” or control centre of this 3-part system). The CGM sends data to the digital controller, which analyzes it and makes decisions about any hormone therapy adjustments needed, and instructs the pump accordingly. Integrating these 3 functions together creates an automated closed-loop system. It is the addition of the digital control function that is the key innovative feature of this new technology.

There are several ways in which APD systems may be classified to order to make sense of the range of development going on in this complex field. We present our findings classified according to the level of automation that each system is capable of, the hormonal approach used, the type of control algorithm used, and the glycemic control strategy used.

The Juvenile Diabetes Research Foundation (now known as JDRF) has defined 6 categories of closed-loop APD technology, based on the level of automation (Figure 1). First generation systems (non-closed-loop; which comprise stages 1-3) have already been mentioned above. Second generation systems, comprising stage 4 and stage 5 systems, are automated insulin-alone delivery (AID) systems. Stage 4 systems are hybrid closed-loop devices, which are closed-loop at all times with mealtime manual assist bolus. Stage 5 systems are fully automated AIDs. Third generation systems (also known as stage 6) are fully automated multihormonal (MH) delivery devices, in which a secondary glucoregulatory hormone such as glucagon or amylin is used in addition to insulin.^4^

**Figure 1.**
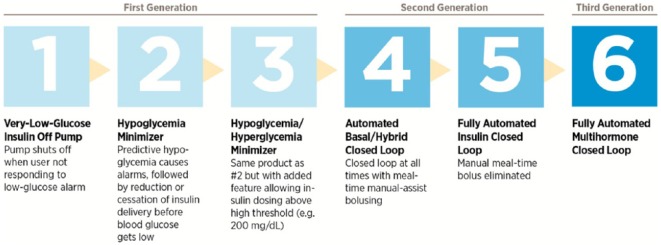
The 6 developmental stages of artificial pancreas device systems (copyright JDRF).^5^

Although the JDRF schema represents stages 1-6 as a linear sequence, it is important to note that all types of closed-loop APDs (stages 4-6) are in fact being developed in parallel. The development of each type does not depend on the previous stage having been completed.^4^

There is much discussion as to the pros and cons of the 2 main hormonal approaches being pursued: AID (also known as insulin-alone/only or single-hormone) and MH (also known as bihormonal or dual-hormone). The inclusion of glucagon in MH systems more closely mimics the way in which blood glucose levels are controlled naturally, as the actions of these 2 hormones are complementary. Although MH control may offer a way of achieving tighter glycemic control and avoiding hypoglycemia, glucagon is unstable in solution and needs to be replaced every 8 hours or so. The other practical issue is that commercial dual infusion pumps need to be developed.

Another way that APD systems can be classified is according to the type of computer algorithm that the digital controller uses. There are 4 main types of control algorithms being used in closed-loop APD systems. Model predictive control (MPC) algorithms predict glucose levels at a specific time point in the near future (some MPC-based systems can “learn” and adapt to the user’s routine and make use of clinician’s input). Proportional integral derivative (PID) algorithms respond to measured glucose levels. Fuzzy logic (FL) algorithms calculate insulin doses based on how a clinical expert would make real-time adjustments based on CGM data. Bio-inspired algorithms are based on a mathematical model of how pancreatic beta cells produce insulin in response to changes in blood glucose levels. In addition to using CGM data, some APD systems measure other biometric/physiological fluctuations (eg, galvanic skin response), and these are known as multivariable or adaptive systems.

The third way of classifying APD systems is according to the strategy that they use for achieving glycemic control, which can be either treat to range (TTR) or treat to target (TTT). TTR means that they aim to keep blood glucose levels within a personalized range, and TTT means they aim to keep them as close as possible to a specific value. These 2 types of glycemic control strategy are also known as control to range (CTR) and control to target (CTT), respectively.

Clinical trials are being conducted in 3 different types of research setting: inpatient settings in which participants are studied using APD systems in a clinic or hospital, outpatient settings in which participants are studied using the systems while they stay for a short time in a specialist diabetes camp or hotel, and in the home setting (with some degree of direct or remote supervision from the research team). In most cases, APD use is being studied over relatively short periods of time (eg, hours, days or weeks), although some studies are looking at longer periods of use (eg, 2-3 months of home use under free-living conditions).^6,7^

## Methods

We defined our inclusion criteria for the horizon scanning review as second and third generation (AID stages 4 and 5; MH stage 6) APD systems in clinical development for the closed-loop control of type 1 diabetes. The review did not include preclinical developments, first generation (stages 1-3) APD systems, or biological types of artificial pancreas technologies, such as implantable bioengineered systems.

To identify relevant technologies, we conducted a predefined, online search of publicly available, restricted access and confidential sources of information about medical devices. These included international horizon scanning technology databases and our own NIHR Horizon Scanning Research & Intelligence Centre’s database, clinical trial registries, bibliographic databases such as MedLine, conference reports and abstracts, review articles and commentaries in specialist journals, and the websites and publications of relevant organizations and developers.

This was supplemented by searching more general sources of information such as Google, health media reports and industry news sites. The search terms used were as follows. Clinical condition: diabetes mellitus type 1, type 1 diabetes. Technology type: artificial pancreas (not bioartificial), bionic pancreas, medical device, medical technology, electromechanical device, closed(closed (-)loop) loop and (diabet* or insulin or glucose), infusion system, insulin and closed-loop system. Clinical role: therapy and control. Phase of research: clinical, human. This research was undertaken between July and September 2014. We then approached the commercial and noncommercial developers identified during the search process to obtain further information about the APD systems we identified. The information was then collated and classified.

## Results

A total of 18 closed-loop APD systems were identified as being in clinical phase development (Table 1). Further information about these systems may be found in the appendix. Six systems were being developed by commercial companies (technologies 1, 3, 5, 10, 11 and 14). Five developers provided approximate timeframes to product launch. It was estimated that 2 systems may become commercially available in the EU in 2016, 1 in 2017, and 2 more in 2018. Timeframe information on the other 11 systems was not available.

**Table 1.** Summary of the Closed-Loop Artificial Pancreas Device (APD) Systems Identified.

Tech no.	Name of system	Commercial developer (where available)	Generation	Stage	Hormonal approach	Control algorithm type	Glycemic control strategy treat to:
Most recent research setting: home							
1	Inreda artificial pancreas	Inreda Diabetic BV	3	6	Insulin + glucagon	PID	Target
2	iLet (Bionic Pancreas)		3	6	Insulin + glucagon	MPC/PD^a^	Target
3	GlucoSitter	DreaMed Diabetes Ltd; Medtronic Diabetes (licensee)	2	4 or 5	Insulin	FL	Target/Range
4	Florence		2	4	Insulin	MPC	Target
5	Diabetes Assistant (DiAs); Including inControl (brand name)	TypeZero Technologies LLC (licensee)	2	4	Insulin	MPC	Target/Range
6	Closed-Loop Glucose-Sensing Insulin-Delivery System		2	5	Insulin	PID	Target^b^
Most recent research setting: outpatient							
7	Closed-Loop Assessment (CLASS)		2/3	5 + 6	Insulin + glucagon	MPC	Target
8	ZMPC		2	5	Insulin	MPC	Range
9	Portable Artificial Pancreas System (pAPS)		2	5	Insulin	MPC/PID/FL	Target/Range
10	Hybrid Closed-Loop (HCL) system (MiniMed 670G insulin pump and Enlite 3 CGM sensor)	Medtronic Diabetes	2	4	Insulin	PID	Target
11	Oregon bihormonal closed-loop system (using Artificial Pancreas Control software)	Legacy Health Systems	3	6	Insulin + glucagon	PID	Range^c^
12	Bio-inspired Artificial Pancreas (BiAP)		2 (→3)^c^	5 (→6)	Insulin + glucagon	Bio-inspired	Target
Research recent setting: inpatient							
13	Integrated Multivariable Adaptive Artificial Pancreas (IMA-AP)		2 (→ 3)	5 (→ 6)	Insulin + glucagon	MPC (GPC)^d^	Target
14	Dose Safety Controller (DSC)	Dose Safety Inc	2	5	Insulin	FL	Target
15	Physiologic Insulin Delivery with Adaptive Basal (PID_AB_)		2	5	Insulin	PID	Target
16	ClosedLoop4Meal Controls (CL4M)		2	5	Insulin	PID	Target
17	Predictive Rule-Based Algorithm (pRBA)		2	4	Insulin	FL	Range
18	DIABELOOP		2	4	Insulin	MPC	Range

a PD, predictive derivative, a form of the PID type control.

b Unconfirmed by developer.

c → capable of.

d GPC, generalized predictive control, a form of MPC-type control.

Twelve systems were classified as second generation (technologies 3-6, 8-10 and 14-18). Of these, 5 were at stage 4 (technologies 4, 5, 10, 17 and 18), 1 was at stage 4/5 (technology 3), and 6 were at stage 5 (technologies 6, 8, 9 and 14-16). Three systems were classified as second/third generation, being at stage 5/6 (technologies 7, 12 and 13). Three systems were classified as third generation, being at stage 6 (technologies 1, 2 and 11). In terms of hormonal approach, 12 systems used the AID (insulin-alone) approach (technologies 3-6, 8-10 and 14-18) and 6 used the MH (insulin and glucagon) approach (technologies 1, 2, 7 and 11-13).

Six systems were based on a PID-type control algorithm (technologies 1, 6, 10, 11, 15 and 16) and one was based on a similar generalized predictive control (GPC) algorithm (technology 13). One system was based on both MPC and predictive derivative (PD) algorithms (technology 2). Five systems were based on an MPC control algorithm (technologies 4, 5, 7, 8 and 18). Three systems were based on a FL control algorithm (technologies 3, 14 and 17). One system was based on PID, MPC and FL control algorithms (technology 9), and 1 system was based on a bio-inspired control algorithm (technology 12). Eleven systems (61%) used a TTT strategy for glycemic control (technologies 1, 2, 4, 6 [not confirmed by the developer], 7, 10 and 12-16), 4 (22%) used a TTR strategy (technologies 8, 11 [not confirmed by the developer], 17 and 18) and 3 (17%) used both (technologies 3, 5 and 9).

Six systems were being tested by users in the home environment (technologies 1-6) and 5 in outpatient settings (technologies 7-11). The remaining 7 APD systems were being tested in inpatient settings (technologies 12-18). Published results from research conducted in the home setting were available for 4 of the APD systems: technology 1,^8^ technology 3,^9,10^ technology 4,^6,11-16^ and technology 5.^7^

## Discussion

We identified 18 APD systems that are in development for the closed-loop control of type 1 diabetes. All of them were being tested in clinical trials, albeit in different settings, some more closely representing a “real-life” situation than others. None of the APD systems are currently available for routine use or are likely to be so before 2016. The first fully automated APD systems are expected to appear on the market in the EU from late 2016 onwards, with around 5 systems expected to be available by the end of 2018. However, their adoption will require appropriate evidence to show that they are safe, and cost and clinically effective, and that they are acceptable to users.

Research to date has shown that APD systems work safely in research settings when used for short periods of time.^17^ During 2014, the first results were published on the use of such systems in outpatient settings including the home.^17^ The research effort is now intensifying to assess effectiveness, with a focus on testing how well these systems work in real-life settings over longer periods of time, and what benefits they may offer.^18^ At present, there is very little detail publically available about the precise functionality of APD systems and the user experience that they may offer, as most systems are still in the developmental phase.

In terms of the technological characteristics of APD systems we found that around two-thirds were following the AID (insulin-alone) hormonal approach and one-third the MH approach (insulin and glucagon). Research using other secondary hormones is also underway funded by the USA National Institutes of Health through Type 1 Diabetes Targeted Research Awards.^19^

MH systems involve added complexities regarding the number of devices that need to be worn and the stability of glucagon. There are different types (and subtype variations) of digital controllers being developed, with some systems incorporating more than 1 type. PID-type control algorithms, which respond to measured glucose levels, are being used in half of the systems. MPC algorithms, which predict glucose levels at a specific time point in the future, are being used in just over one-third. FL algorithms, which model clinical decision-making, are being used in around a fifth of systems. A bio-inspired control algorithm which models how pancreatic beta cells work is being used in 1 system.

APD systems are using different strategies for achieving glycemic control, with 61% using the TTT strategy, 22% using the TTR strategy, and 17% using both.

How an APD system looks and feels from the users’ perspective will be vitally important.^20^ There are various ways in which the 3 key functions of an APD (to monitor, control and treat) might be configured in commercial products. First, the control function could be integrated into the pump or CGM device, with a smartphone or smartwatch app providing the user interface with the controller. In this configuration, no separate device would need to be carried to make the transition from CGM/pump-use to APD if the user routinely carried a smartphone or smartwatch.

Second, the control function could be performed by a separate mobile controller device connected wirelessly to the CGM and pump. The controller would be carried by the user whilst the CGM and pump are attached to the body. The controller device would look similar to a modified smartphone, but would only function for this purpose. In this configuration, an additional device would need to be carried to make the transition from CGM/pump-use to APD system. If 2 pumps or a second backup CGM are needed then other devices would be needed.

Third, the control function could be in the form of an app installed on a regular smartphone or smartwatch. The APD control app would run alongside all the other functions performed by such technologies. In this configuration, no separate device would need to be carried to make the transition from CGM/pump-use to APD system if the user usually carried a smartphone or smartwatch.

## Conclusions

We found that 18 closed-loop APD systems are being developed through clinical stages of research, and these employ different combinations of hormonal approaches, control algorithms and glycemic control strategies. However, there is currently little information publically available on precisely what form they might take as commercial products. APD systems have so far been tested in small-scale clinical trials over relatively short periods of time. Further phase 3 clinical research will be needed to demonstrate that they are safe, and clinically and cost-effective for long-term 24/7 use. For this new form of technology to be adopted successfully, developers would also need to ensure that their products meet user expectations in terms of design, functionality and impact on quality of life.

## Appendix

**Appendix:** Further Information on the Closed-Loop Artificial Pancreas Device (APD) Systems Identified

No.	Name of APD system	Developer	Technology description
1	Inreda artificial pancreas	Inreda Diabetic BV	A bihormonal system that is waterproof and Wi-Fi connected. According to the developer’s website this device provides a fully autonomous regulation of glucose levels, and is expected to reach the market in 2016. All the components are integrated into a system comprised of 2 pumps (one each for insulin and glucagon), 2 CGMs, Wi-Fi, sensoring, alarm sets, and 2 batteries. The system uses an intelligent reactive control algorithm that works out when and how much insulin or glucagon needs to be administered. Insulin delivery is determined by the difference between current and target glucose levels, glucose rate of change, the insulin sensitivity of the user, and 2 glucose thresholds, which triggers the delivery of a corrective insulin bolus. The controller uses AA batteries and transmits the data to a database every 24 hours. Audible alarms alert users if they need to check something or to take action. Intended use: continuous use in daily life for adults. Recent published research.^22^ Ongoing and planned research (using a prototype market model): NCT02160275. Further information: http://www.inredadiabetic.nl/
2	iLet (Bionic Pancreas)	USA research team: Massachusetts General Hospital and Boston University	A fully integrated and automated bihormonal closed-loop system. According to the developer’s website, clinical trials of the iLet are expected to begin in mid-2016. The system has a built-in wireless CGM that works with a Dexcom sensor and transmitter, and a dedicated handheld controller device on which its dosing algorithms run. The control algorithms “learn” about and adapt to the user’s insulin requirements, enabling personalized management. The system also contains 2 independent pumps which are automatically commanded by the insulin and glucagon dosing algorithms every 5 minutes. The bionic pancreas is being tested in the home setting. Recent published research.^23,24^ Completed research: NCT02105324, NCT02092220. Ongoing or planned research: NCT02105324, NCT01833988, NCT01762059, NCT02181127, NCT02509065. Further information: http://sites.bu.edu/bionicpancreas/
3	GlucoSitter Based on the MD-Logic algorithm (MDLAP)	DreaMed Diabetes Ltd; Medtronic Diabetes (licensee)	An automated system that analyzes input data from a sensor and commands the pump to deliver the correct dose of insulin at the right time. It is based on the MD-Logic algorithm (FL type), which is intended to be either integrated within an insulin pump or operated on a dedicated handheld device. It has an alert based real-time pump and sensor data, and embedded insulin delivery safety layers. It uses a conventional basal/bolus pump therapy approach, and the user can transition between closed-loop and manual control. Intended use: daytime or overnight; all age groups. In Phase 3 clinical research. Recent published research.^25-30^ Ongoing and planned research: NCT01238406, NCT01901913, NCT01726829, NCT01308164, NCT01157923. An overnight version of the GlucoSitter algorithm has been CE marked. Further information: http://www.dreamed-diabetes.com/; http://diatribe.org/first-ever-artificial-pancreas-software-receives-european-approval
4	Florence	UK research team: Cambridge University (Cambridge Enterprise). Part of the JDRF Artificial Pancreas Project (APP) Consortium and AP@home consortium	The latest research version (FlorenceM) consists of a next generation sensor augmented Medtronic 640G insulin pump and Medtronic Enlite 3 family real-time CGM with glucose suspend feature, and an Android smartphone containing the Cambridge MPC control algorithm. The controller communicates wirelessly with the pump using a proprietary translator device. Recent published research.^31-37^ Planned research: NCT02523131. Further information: http://www.cam.ac.uk/research/news/new-study-shows-artificial-pancreas-works-for-length-of-entire-school-term
5	Diabetes Assistant (DiAs); including inControl (brand name)	TypeZero Technologies LLC (licensee); international research teams: USA (Sansum Research Institute, Santa Barbara, California, Universities of Virginia and Stanford), France (Montpellier), Netherlands (Amsterdam), Israel (Petah Tikva) and Italy (Padova)	DiAs is a modular artificial pancreas platform based on an Android smartphone. The smartphone app contains the computer program that controls blood glucose (including predicting when levels will rise and fall). It wirelessly receives data from the CGM (Dexcom G4) and gives commands via Bluetooth to an insulin pump (eg, Tandem t:slim or Accu-Check from Roche). There is also a special Bluetooth box that connects to local data servers and the Cloud. The controller is compatible with any CGM or insulin pump. The system is being tested in long-term (1-3 months) clinical trials in the home setting. TypeZero has developed a smartphone-based APD called inControl, a mobile-based advisory system called inControl Advice that generates real-time recommendations for meals, basal rates, bolus calculations and exercise decisions, and a cloud-based analytics and support system called inControl Cloud (which enables real-time monitoring and notifications for caregivers, and retrospective analysis of data). Intended use: daytime, night time, during exercise; children and adult. Recent published research.^38-41^ Ongoing and planned research (including the use of the ZMPC algorithm on the DiAs platform): NCT02463682, NCT02514785, NCT02506764, NCT02302963. Further information: http://www.medpagetoday.com/Blogs/DiabetesDiscovery/51124; http://typezero.com/; http://www.healthline.com/diabetesmine/typezero-tech-closed-loop-commercialization#1
6	Closed-Loop Glucose-Sensing Insulin-Delivery System (bionic pancreas)	Australian research team: St Vincent’s Hospital, Melbourne. Part of the JDRF APP Consortium	Closed-Loop Glucose-Sensing Insulin-Delivery System using a controller device based on a modified smartphone. The Android device processes information from the CGM and uses it to control the delivery of insulin. Intended use: overnight or as a 24-hour hybrid closed-loop system. Ongoing research: NCT02040571.
7	Closed-Loop Assessment (CLASS)	Canadian research team: Montreal University, Institut de Recherches Cliniques de Montreal and McGill University. Part of the JDRF APP Consortium	Regulates glucose levels automatically, adjusts insulin delivery every 10 minutes and sometimes gives glucagon boluses in response to falling glucose levels. The glucagon boluses are small in size, leading to a total daily glucagon dose of (in most cases) less than 20% of that used to treat severe hypoglycemia. The system gives meal boluses; the user inputs a description of each meal as being either small, medium or large. Intended use: day and night, exercise; adults and children. In phase 2 and phase 3 clinical research. Recent published research.^42,43^ Completed research: NCT01930110, NCT01966393. Ongoing and planned research: NCT02416765, NCT02282254, NCT02488616, NCT02490098.
8	ZMPC (Zone-Model Predictive Control)	USA and Italian research teams: USA (Sansum Diabetes Research Institute and Universities of California Santa Barbara and Virginia) and Italy (University of Padova). Part of the JDRF APP Consortium	Uses an algorithm described as being the next generation in MPC-type control models. The system uses a dynamic model that relates the effect of subcutaneously injected insulin on blood glucose concentration and uses this to make predictions into the near future. For a given desired glucose target zone, the model can be used to calculate how much insulin needs to be delivered in the future to maintain glucose levels within target range. The instruction for the first of these insulin amounts is sent to the insulin pump which delivers it to the user, and the calculations are repeated when new information becomes available (ie, a new CGM measurement comes in). Intended use: 24+ hours (including exercise), all age groups. In phase 2 clinical research. Recent published research.^44^ Ongoing and planned research (using the ZPMC algorithm on the DiAs platform): NCT02463682, NCT02514785, NCT02506764. Further information: http://www.news.ucsb.edu/2015/014982/pediatric-diabetes-gamechanger
9	portable Artificial Pancreas System (pAPS)	USA research team: Sansum Diabetes Research Institute and University of California at Santa Barbara. Part of the JDRF APP Consortium	Consists of an APD platform in a portable computer. The system connects a subcutaneous CGM and a subcutaneous insulin delivery pump for automated adjustment of insulin dosing. Intended use: 24+ hours (ongoing), including exercise, for adults. In phase 2 clinical research. Recent published research.^45-47^
10	Hybrid Closed-Loop (HCL) system (MiniMed 670G insulin pump and Enlite 3 CGM sensor). Versions of this system have been known by the following names: OCL (Overnight Closed-Loop System) and ePID (MiniMed external physiological insulin delivery) system	Medtronic Diabetes; USA, UK, and Israeli research teams: USA (University of Yale), UK (Kings College London) and Israel (Sheba Medical Centre). Part of the JDRF APP Consortium	Threshold suspend stops insulin delivery in a particular situation (when the sensor glucose reaches a preset low threshold), and the system also continually measures and adjusts insulin delivery rates based on sensor glucose readings. The user would need to enter their carbohydrates at mealtime and calibrate the sensor periodically. Intended use: to automatically control glucose levels 24/7. In phase 3 clinical research: NCT02463097. According to media reports commercial launch is expected in April 2017 for the USA and April 2018 for the international market.^48^ Further information: http://newsroom.medtronic.com/phoenix.zhtml?c=251324&p=irol-newsArticle&ID=2056959
11	Oregon bihormonal closed-loop system (using Artificial Pancreas Control software)	Legacy Health Systems; USA research team: Oregon Health & Science University and Rensselaer Polytechnic Institute	A bihormonal system comprised of 2 CGMs, 2 pumps, and a custom made battery pack. The control software runs on a smartphone device, which connects wirelessly with the other components. It is capable of delivering both a premeal insulin bolus and automated insulin or glucagon infusion. The indirect adaptive proportional-derivative (PID-type) controller works in concert with a mathematical model of glucose regulation to enable it to adjust for changes in insulin sensitivity. In phase 2 clinical research. Recent published research.^49,50^ Completed research: NCT01552603. Further information: http://www.rpi.edu/innovation/InnovationSpring2015/feature-pancreas.html
12	Bio-inspired Artificial Pancreas (BiAP)	UK research team: Imperial College London	This bio-inspired system differs from other closed-loop systems in that the control algorithm is based on a mathematical model of the glucose responses of biological alpha and beta cells in the pancreas providing physiological control. It is also fully implemented on a low-power miniature silicon microchip. The BiAP system is comprised of a microchip (that reproduces physiological insulin and glucagon release), a commercial continuous glucose sensor, and continuous subcutaneous infusion pumps to provide bihormonal glucose control. In research studies an Enlite sensor (Medtronic) and Accu-Chek insulin pump (Roche) have been used, although the handheld unit could be integrated with any commercially available sensors and pumps. Intended use: throughout the day (including exercise and other activities of daily living) for adults. Recent published research.^51-53^ In phase 2 clinical research, with an unsupervised home study underway: NCT02397265. Further information: http://www3.imperial.ac.uk/bioinspiredtechnology/research/bionicpancreas
13	Integrated Multivariable Adaptive Artificial Pancreas (IMA-AP)	USA and Canadian research teams: USA (Illinois Institute of Technology, University of Illinois at Chicago, University of Chicago) and Canada (York University, Toronto). Part of the JDRF APP Consortium	Uses a generalized predictive control (GPC) algorithm plus a hypoglycemia early alarm system. Both systems use recursive models updated at each sampling time. Subcutaneous glucose concentrations and several physiological signals from a sports armband are collected and used in glucose prediction models and control algorithms. Intended use: day and night, 24/7. Recent published research.^54-56^ Further information: http://www.iit.edu/ecdre/research/new_awards.shtml; https://www.facebook.com/pages/Multivariable-Artificial-Pancreas
14	Dose Safety Controller (DSC)	Dose Safety Inc	This is a TTT, insulin-only system which can switch between 3 modes of operation: (a) auto-dosing (the default mode), (b) suspended, and (c) disabled. In auto-dosing mode, the insulin pump only infuses dose amounts calculated by the Fuzzy Logic Dosing Module (FLDM). Auto-dosing provides all of the user’s insulin needs: after meals and during fasting periods. According to the developer’s website, DSC software runs on any smartphone or programmable chip in an insulin pump. Intended use: fully automated 24/7. In late stage feasibility studies. Recent published research.^57^ Further information: www.dosesafety.com
15	Physiologic Insulin Delivery with Adaptive Basal (PID_AB_)	USA research team: Boston Children’s Hospital and Harvard Medical School. Part of the JDRF APP Consortium	Includes a meal identification algorithm designed to allow the insulin normally used to cover a meal to be delivered within the first 15-30 minutes of a meal, with more insulin being delivered within 30-45 minutes of the meal and approximately the same amount delivered by 60 minutes. It includes an adaptive basal adjustment which allows changes in basal rate, similar to those observed in pump users to be affected over a 30 minute interval with limited hypo or hyperglycemic excursions (target ±15 mg/dL). Intended use: 24 hour, all ages. Recent published research.^58^
16	ClosedLoop 4Meal Controls (CL4M)	Spanish research team: Hospital Clinic of Barcelona, Hospital Clinic of Valencia, Universidad Politécnica de Valencia and Universidad de Girona	Includes a safety auxiliary feedback based on sliding mode reference conditioning, which aims to limit over-dosing on insulin. It follows a 2-stage approximation to constrained control, and is claimed to be sensitive to sensor failures. The algorithm, called “SAFE,” is a security loop to be added to the main control loop. In phase 1 clinical research: NCT02100488. Further information: http://closedloop4meal.org/
17	Predictive Rule-Based Algorithm (pRBA)	Spanish research team: Autonomous University of Madrid and Hospital de Sabadell	A hybrid APD system that calculates the amount of insulin needed every 5 minutes using CGM values, information on daily events such as carbohydrate intake, and its own prediction of glucose levels. Each system is individualized to its user, based on their body weight and sensitivity to insulin. Intended use: 24 hours (including exercise). In phase 1 clinical research: NCT02160184. Recent published research.^59^
18	DIABELOOP	French research team: CERITD, Grenoble	The system comprises an insulin pump, a glucose sensor, a controller (a restricted smartphone where the MPC algorithm software and interface resides), a remote server with alert algorithms, an interface for the medical team and user support service. It has the potential for user information input (eg, meals and physical activity). Intended use: 24 hour. In phase 2 clinical research. Recent published research.^60,61^ Completed research: NCT02101229, NCT01754181, NCT01640210, NCT01640223. Further information: www.diabeloop.fr; http://www.biovision.org/pdf/projects/Diabeloop.pdf

Source: NIHR Horizon Scanning Research & Intelligence Centre.^21^
